# Biological and Biochemical Characterization of Mice Expressing Prion Protein Devoid of the Octapeptide Repeat Region after Infection with Prions

**DOI:** 10.1371/journal.pone.0043540

**Published:** 2012-08-21

**Authors:** Yoshitaka Yamaguchi, Hironori Miyata, Keiji Uchiyama, Akira Ootsuyama, Sachiko Inubushi, Tsuyoshi Mori, Naomi Muramatsu, Shigeru Katamine, Suehiro Sakaguchi

**Affiliations:** 1 Division of Molecular Neurobiology, The Institute for Enzyme Research (KOSOKEN), The University of Tokushima, Tokushima, Japan; 2 Department of Molecular Microbiology and Immunology, Nagasaki University Graduate School of Biomedical Sciences, Nagasaki, Japan; 3 Animal Research Center, School of Medicine, University of Occupational and Environmental Health, Kitakyushu, Japan; 4 Department of Radiation Biology and Health, School of Medicine, University of Occupational and Environmental Health, Kitakyushu, Japan; Ohio State University, United States of America

## Abstract

Accumulating lines of evidence indicate that the N-terminal domain of prion protein (PrP) is involved in prion susceptibility in mice. In this study, to investigate the role of the octapeptide repeat (OR) region alone in the N-terminal domain for the susceptibility and pathogenesis of prion disease, we intracerebrally inoculated RML scrapie prions into tg(PrPΔOR)/*Prnp^0/0^* mice, which express mouse PrP missing only the OR region on the PrP-null background. Incubation times of these mice were not extended. Protease-resistant PrPΔOR, or PrP^Sc^ΔOR, was easily detectable but lower in the brains of these mice, compared to that in control wild-type mice. Consistently, prion titers were slightly lower and astrogliosis was milder in their brains. However, in their spinal cords, PrP^Sc^ΔOR and prion titers were abundant and astrogliosis was as strong as in control wild-type mice. These results indicate that the role of the OR region in prion susceptibility and pathogenesis of the disease is limited. We also found that the PrP^Sc^ΔOR, including the pre-OR residues 23–50, was unusually protease-resistant, indicating that deletion of the OR region could cause structural changes to the pre-OR region upon prion infection, leading to formation of a protease-resistant structure for the pre-OR region.

## Introduction

Transmissible spongiform encephalopathies or prion diseases, which include Creutzfeldt-Jakob disease in humans and scrapie and bovine spongiform encephalopathy in animals, are neurodegenerative disorders caused by prions [1], [2]. Prions consist mainly of the abnormally folded, proteinase K (PK)-resistant isoform of prion protein, designated PrP^Sc^ [3]. Structural conversion of the normal cellular isoform, designated PrP^C^, into PrP^Sc^ is a key event in prion propagation. Indeed, mice devoid of PrP^C^ (*Prnp^0/0^*) are resistant to the disease without PrP^Sc^ accumulation and prion propagation in the brain, even after inoculation with prions [4], [5], [6], [7]. However, the exact conversion mechanism remains largely unknown.

PrP^C^ is a glycoprotein tethered to the outer cell membrane via a glycosylphosphatidylinositol anchor moiety and expressed most abundantly in the brain, particularly by neurons [8]. Reduction in susceptibility to RML scrapie prions was reported in tg(PrPΔ32–93)/*Prnp^0/0^* and tg(PrPΔ23–88)/*Prnp^0/0^* mice, which express mouse (mo) PrP lacking residues 32–93 or 23–88 on the *Prnp^0/0^* background, respectively [9], [10]. The incubation times of these mice were accordingly extended [9], [10]. The incubation times of experimental prion diseases in mice are usually inversely correlated to the expression level of PrP^C^ in the brain. Indeed, tg(moPrP)/*Prnp^0/0^* mice, which express mouse wild-type PrP^C^ in the brains at 8 fold higher levels than control wild-type mice, showed a shorter incubation time of 50±2 days post-inoculation (dpi) with RML prions, while the wild-type mice became sick at 127±1 dpi [10], [11]. Tg(PrPΔ23–88)/*Prnp^0/0^* mice were shown to express PrPΔ23–88 in their brains two fold higher than moPrP^C^ in tg(moPrP)/*Prnp^0/0^* mice [10]. However, tg(PrPΔ23–88)/*Prnp^0/0^* mice developed the disease with a longer incubation time of 161±4 dpi than tg(moPrP)/*Prnp^0/0^* mice with 50±2 dpi [10]. Tg(PrPΔ32–93)/*Prnp^0/0^* mice also developed the disease with longer incubation times of 232 to 313 dpi than control wild-type mice with 158±11 dpi, although tg(PrPΔ32–93)/*Prnp^0/0^* mice expressed PrPΔ32–93 in the brains 4 fold higher than PrP^C^ in the control mice [9]. These results indicate that the N-terminal residues of PrP affect susceptibility to RML prions in mice. It was also reported that the MHM2(Δ23–88) molecule, a mouse-hamster chimeric PrP deletion mutant carrying hamster PrP-derived methionine residues at 108 and 111 substituted for leucine and valine residues in mouse PrPΔ23–88, completely failed to restore susceptibility to RML prions in *Prnp^0/0^* mice [10], [11]. These results indicate that the chimeric region, corresponding to residues 108 through 111, also influences the susceptibility to RML prions in mice.

The so-called octapeptide repeat (OR) region, which comprises 5 copies of an octapeptide sequence, is located in the unstructured N-terminal domain of PrP. PrPΔ32–93 lacks the entire OR region (residues 51–90) and most of the OR region is missing in PrPΔ23–88. It is thus suggested that the OR region might be involved in the susceptibility to RML prions in mice. However, PrPΔ32–93 and PrPΔ23–88 lack not only the OR region but also other regions. Therefore, it still remains unclear whether the decreased susceptibility in tg(PrPΔ32–93)/*Prnp^0/0^* and tg(PrPΔ23–88)/*Prnp^0/0^* mice could be due to the deletion of the OR region either alone or together with other regions.

Unusual phenotypes were reported in infected tg(PrPΔ32–93)/*Prnp^0/0^* mice. PrP^Sc^Δ32–93 was hardly detectable in the brains of terminally ill tg(PrPΔ32–93)/*Prnp^0/0^* mice [9]. Prion infectivity was accordingly reduced and disease-specific vacculoation and astrogliosis were undetectable in their brains [9]. However, in the spinal cord, prion infectivity and the pathological changes were similarly observed between tg(PrPΔ32–93)/*Prnp^0/0^* and control mice [9]. Infected tg(PrPΔ32–93)/*Prnp^0/0^* mice also displayed the unusual symptom of foreleg paresis [9]. In contrast, no such unusual phenotypes were detected in infected tg(PrPΔ23–88)/*Prnp^0/0^* mice. Residues 89–93 are missing in PrPΔ32–93, but not in PrPΔ23–88. Therefore, deletion of these residues might be involved in development of the unusual phenotypes, as observed in infected tg(PrPΔ32–93)/*Prnp^0/0^* mice. However, this possibility still remains to be clarified.

We previously established a tg mouse line, designated tg(PrPΔOR)/*Prnp^0/0^*, which expresses mouse PrP with a deletion of the OR region alone on the *Prnp^0/0^* background [12]. In the present study, to investigate the role of the OR region alone in prion susceptibility and the pathogenesis of prion disease, we intracerebrally inoculated RML prions into tg(PrPΔOR)/*Prnp^0/0^* mice.

## Materials and Methods

### Ethics Statement

The Ethics Committee of Animal Care and Experimentation of University of Occupational and Environmental Health, Kitakyushu, Japan approved this study (approval number AE-080-13). Animals were cared for in accordance with The Guiding Principle for Animal Care and Experimentation of University of Occupational and Environmental Health and Japanese Law for Animal Welfare and Care.

### Animals

C57BL/6 mice were purchased from CLEA Japan, Tokyo, Japan and ddY mice were from Kyudo, Tosu, Japan. ddY mice are outbred albino mice maintained in a closed colony. Tg(PrPΔOR)/*Prnp^0/0^* mice with the C57BL/6×129Sv×FVB mixed background were produced elsewhere [12]. In this study, tg(PrPΔOR)/*Prnp^0/0^* mice (C57BL/6×129Sv×FVB) were crossed at least more than twice with Zrch I *Prnp^0/0^* mice, which had been backcrossed to C57BL/6 mice more than 9 times.

### Prion Inoculation

Brains were removed from terminally ill wild-type C57BL/6 mice infected with RML prions. A single brain was homogenized (10%, w/v) in phosphate-buffered saline (PBS) by passing it through 18 to 26 gauge needles and then diluted to 1% with PBS. Four to five week-old mice were intracerebrally inoculated with a 20 µl-aliquot of the homogenates.

### Western Blotting

Tissue homogenates (10%, w/v) were prepared in lysis buffer containing 150 mM NaCl, 50 mM Tris-HCl (pH 7.5), 0.5% Triton X-100, 0.5% sodium deoxycholate, 1 mM EDTA, and protease inhibitor mixture (Nakalai Tesque Co., Kyoto, Japan) by passing them through 18 to 26 gauge needles and centrifugation at low speed to remove debris. Protein concentrations of the resulting supernatant were determined using the BCA protein assay kit (Pierce, Rockford, USA.). Total proteins treated with or without PK (Wako Pure Chemical Industries, Ltd., Osaka, Japan) at 20 µg/ml for 30 min at 37°C were electrophoresed through a 12% SDS-polyacrylamide gel and electrically transferred to an Immobilon-P PVDF membrane (Millipore Corp., MA, USA). The membrane was immersed in 5% non-fat dry milk-containing TBST (0.1% Tween-20, 100 mM NaCl, 10 mM Tris-HCl, pH 7.6) for 1 h at room temperature (RT), and incubated with SAF32 and SAF61 mouse monoclonal antibodies (SPI-BIO, Montigny le Bretonneux, France), M20 goat polyclonal antibodies (Santa Cruz Biotechnology, Inc., Santa Cruz, CA), IBL-N rabbit polyclonal antibodies (Immuno Biological Laboratories, Gunma, Japan), 3F4 monoclonal antibody (Signet Laboratories Inc., Dedham, MA), anti-human glial fibrillary acidic protein (GFAP) IgG rabbit polyclonal antibodies (SHIMA Laboratories Co., LTD, Tokyo, Japan) or anti-β-actin monoclonal antibody (Sigma-Aldrich, Inc., St. Louis, MO) for 2 h at RT or overnight at 4°C in 1% non-fat dry milk-containing TBST. The membrane was washed in TBST for 15 min once and for 5 min three times. Signals were visualized using horseradish peroxidase (HRP)-conjugated anti-mouse IgG antibodies (Amersham Biosciences Inc., Piscataway, NJ), anti-rabbit IgG antibodies (Amersham Biosciences Inc.), and anti-goat IgG antibodies (CHEMICON International, Inc., Temecula, CA) and Immobilon™ Western Chemiluminesent HRP substrate (Millipore) and detected using a chemiluminescence image analyzer, LAS-4000 mini (Fujifilm Co., Tokyo, Japan).

### Immunohistochemistry

Paraffin embedded samples were sectioned, deparaffinized, rehydrated and treated with L.A.B. Solution (Polysciences, Inc., U.S.A.) for 10 min. Nonspecific endogenous peroxidase activity was quenched by incubating the specimens with 3% H_2_O_2_ for 10 min and then the specimens were blocked with 5% normal rabbit serum for 10 min at RT. For detection of PrP^Sc^ or PrP^Sc^ΔOR, the specimens were treated with formic acid for 1 min before the blocking step. The specimens were then incubated with 1/500 polyclonal rabbit anti-GFAP antibodies (DAKO Cytomation, Denmark) or 1/100 polyclonal rabbit IBL-N anti-PrP antibodies (Immuno Biological Laboratories) for 2 h at RT. After washing in PBS, the specimens were incubated with HRP-labeled polymer anti-rabbit (EnVision™ System, DAKO Cytomation, Denmark) for 1 h at RT, washed in PBS, and then visualized using the avidin-biotin complex method (Vector Labs, U.S.A.). The nuclei were counterstained with Mayer’s hematoxylin.

### PNGase F Digestion

PNGase F digestion was performed according to the manufacturer’s protocol (New England Biolabs, Inc., Ipswich, MA). Briefly, the PK-treated homogenates were denatured by boiling for 10 min in the presence of 0.5% SDS and 1% mercaptoethanol and then treated with PNGase F (500 units/L) in 1% Nonidet P-40 and 0.05 M sodium phosphate (pH 7.5) for 60 min at 37°C.

### Standard Curve and Prion Titer Determination

To create a standard curve between prion titers and incubation times, 10% (w/v) brain homogenate of RML-infected ddY mice were serially diluted 10-fold with PBS, ranging from 10^−1^ to 10^−10^ in PBS, and a 20 µl-aliquot of each dilution was intracerebrally inoculated into ddY mice aged 4–5 weeks. The mice were observed until 1 year after inoculation. The ID_50_/gram of the tissue was determined according to the method of Reed and Muench and then a standard curve was created. Prions titers (ID_50_/g) in tissues of interest were determined as follows: A 20 µl-aliquot of the tissue homogenates was intracerebrally inoculated into 5 or 6 ddY mice aged 4–5 weeks and their incubation times were determined. Thereafter, prion titers in the homogenates were calculated using the standard curve.

### Expression Vectors

To construct an expression vector encoding mouse PrP tagged with the 3F4 epitope designated moPrP(3F4), the 5′ fragment of mouse PrP cDNA was amplified by polymerase chain reaction (PCR) using a mouse PrP cDNA as a template with a BamHI-PrP(ATG)-S sense primer (Table S1) and a moPrP-3F4 anti-sense primer (Table S1). Then, full-length PrP cDNA was amplified by PCR using a mouse PrP cDNA as a template with the amplified 5′ fragment as a sense primer and a PrP(stop)-XbaI-AS anti-sense primer (Table S1). After sequence confirmation, the amplified fragment was inserted into *Bam*H I/*Xba* I-digested pcDNA3.1(+) (Invitrogen, Carlsbad, CA), resulting in pcDNA3.1-moPrP(3F4).

To construct an expression vector encoding the 3F4-tagged mouse PrP with a deletion of residues 32–88, designated moPrP(3F4) Δ32–88, the 5′ fragment of moPrP(3F4) Δ32–88 cDNA was amplified by PCR using pcDNA3.1-moPrP(3F4) as a template with a BamHI-PrP(ATG)-S sense primer and a PrP32–88 anti-sense primer (Table S1). Then, full-length moPrP(3F4) Δ32–88 cDNA was amplified by PCR using pcDNA3.1-moPrP(3F4) as a template with the amplified 5′ fragment as a sense primer and a PrP(stop)-XbaI-AS anti-sense primer. After sequence confirmation, the amplified fragment was inserted into *Bam*H I/*Xba* I-digested pcDNA3.1(+) (Invitrogen, Carlsbad, CA), resulting in pcDNA3.1-moPrP(3F4)32–88.

To construct expression vectors encoding moPrP(3F4) Δ32–88(3K3A), moPrP(3F4) Δ32–88(K23A), moPrP(3F4) Δ32–88(K24A), moPrP(3F4) Δ32–88(K27A), moPrP(3F4) Δ32–88(K23,24A), moPrP(3F4) Δ32–88(K23,27A), moPrP(3F4) Δ32–88(K24,27A), moPrP(3F4) Δ32–88(3K3R), moPrP(3F4) Δ32–88(2P2A), moPrP(3F4) Δ32–88(2P2G) and moPrP(3F4) Δ32–88(2P2W), the 5′ fragment of the moPrP(3F4) Δ32–88 vector was amplified by PCR using a vector-derived T7 sense primer (Table S1) and an antisense primer (Table S1) of PrP(3K3A)-AS, PrP(K23A)-AS, PrP(K24A)-AS, PrP(K27A)-AS, PrP(K23/24A)-AS, PrP(K23/27A)-AS, PrP(K24/27A)-AS, PrP(3K3R)-AS, PrP(2P2A)-AS, PrP(2P2G)-AS or PrP(2P2W)-AS, respectively. The amplified fragments were then used as sense primers for amplification of full-length cDNAs encoding each mutant PrP with a vector-derived BGH reverse primer (Table S1) as an antisense primer using the moPrP(3F4) Δ32–88 vector as a template. After sequence confirmation, each amplified fragment was inserted into *Bam*H I/*Xba* I-digested pcDNA3.1(+) (Invitrogen).

### Transfection

Mouse neuroblastoma N2a cells persistently infected with 22L prions, designated N2aC24L1-3 [13], were transiently transfected with expression vectors using Lipofectamine 2000 reagent (Invitrogen). The cells were lysed in a buffer (150 mM NaCl, 0.5% Triton X-100, 0.5% sodium deoxycholate, 50 mM Tris-HCl, pH 7.5) 2 days after transfection and subjected to Western blotting.

### Statistical Analysis

Log-rank test was used for analysis of the incubation times of infected mice.

## Results

### Incubation Times and Foreleg Paresis in tg(PrPΔOR)/*Prnp^0/0^* Mice after Infection with RML Prions

**Figure 1:**
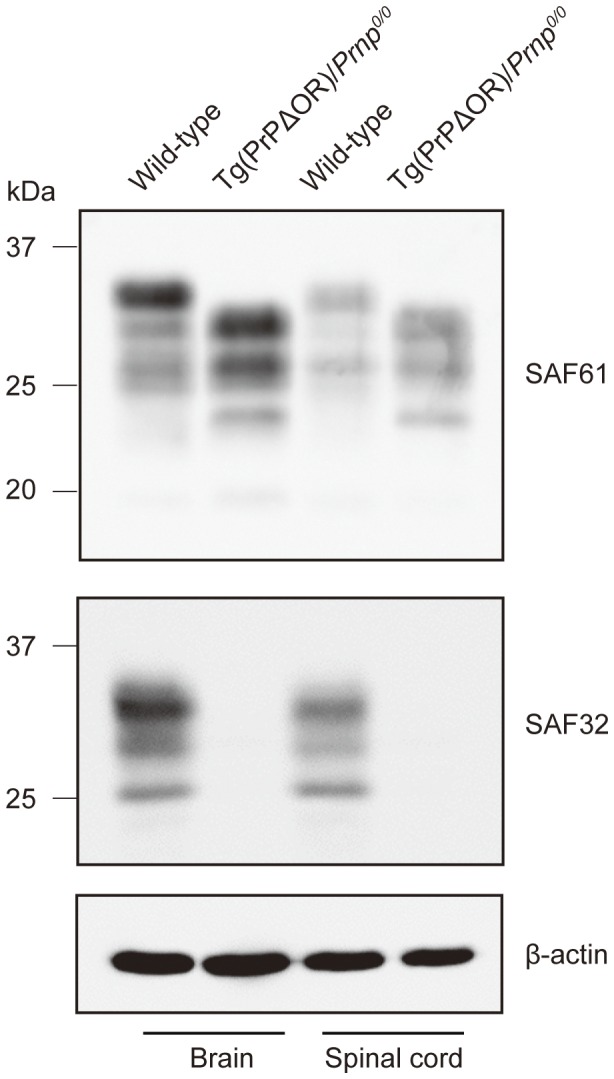
Overexpression of PrPΔOR in the brains and spinal cords of tg(PrPΔOR)/*Prnp^0/0^* mice. The brain and spinal cord homogenates from tg(PrPΔOR)/*Prnp^0/0^* mice and wild-type mice were subjected to Western blotting with SAF61 or SAF32 anti-PrP antibodies. The expression of β-actin was detected in these homogenates as an internal control.

We intracerebrally inoculated RML prions into tg(PrPΔOR)/*Prnp^0/0^* mice and control C57BL/6 wild-type mice. Uninfected tg(PrPΔOR)/*Prnp^0/0^* mice remained healthy for more than 500 days. Wild-type mice developed disease-specific symptoms, such as weight loss, decreased locomotive activity, ruffled hair coat and hunched back, at 165±4 days post-inoculation (dpi) (Table 1). Tg(PrPΔOR)/*Prnp^0/0^* mice succumbed to the disease with slightly shorter incubation times of 147±9 dpi (Table 1). This is probably due to higher expression of PrPΔOR in the brains of tg(PrPΔOR)/*Prnp^0/0^* mice than in that of PrP^C^ in wild-type mice. PrPΔOR was detected in the brain and spinal cord about 2–3 fold more than PrP^C^ in wild-type mice on Western blotting using SAF61 anti-PrP antibodies, which recognize residues 142–160 (human PrP numbering) (Fig. 1). Lack of the OR region in PrPΔOR was confirmed by Western blotting using SAF32 anti-OR region antibody (Fig. 1). Tg(PrPΔOR)/*Prnp^0/0^* mice also displayed the additional unusual symptom of foreleg paresis at early stages of the disease.

**Table 1:** Incubation times in tg(PrPΔOR)/*Prnp^0/0^* and wild-type mice after infection with RML prions.

Mouse line	Incubation times (Mean±SD, days)	Diseased/Inoculated
Wild-type (C57BL/6)	165±4	10/10
Tg(PrPΔOR)/*Prnp0/0*	147±9	8/8

### Astrogliosis in tg(PrPΔOR)/*Prnp^0/0^* Mice Infected with RML Prions

**Figure 2:**
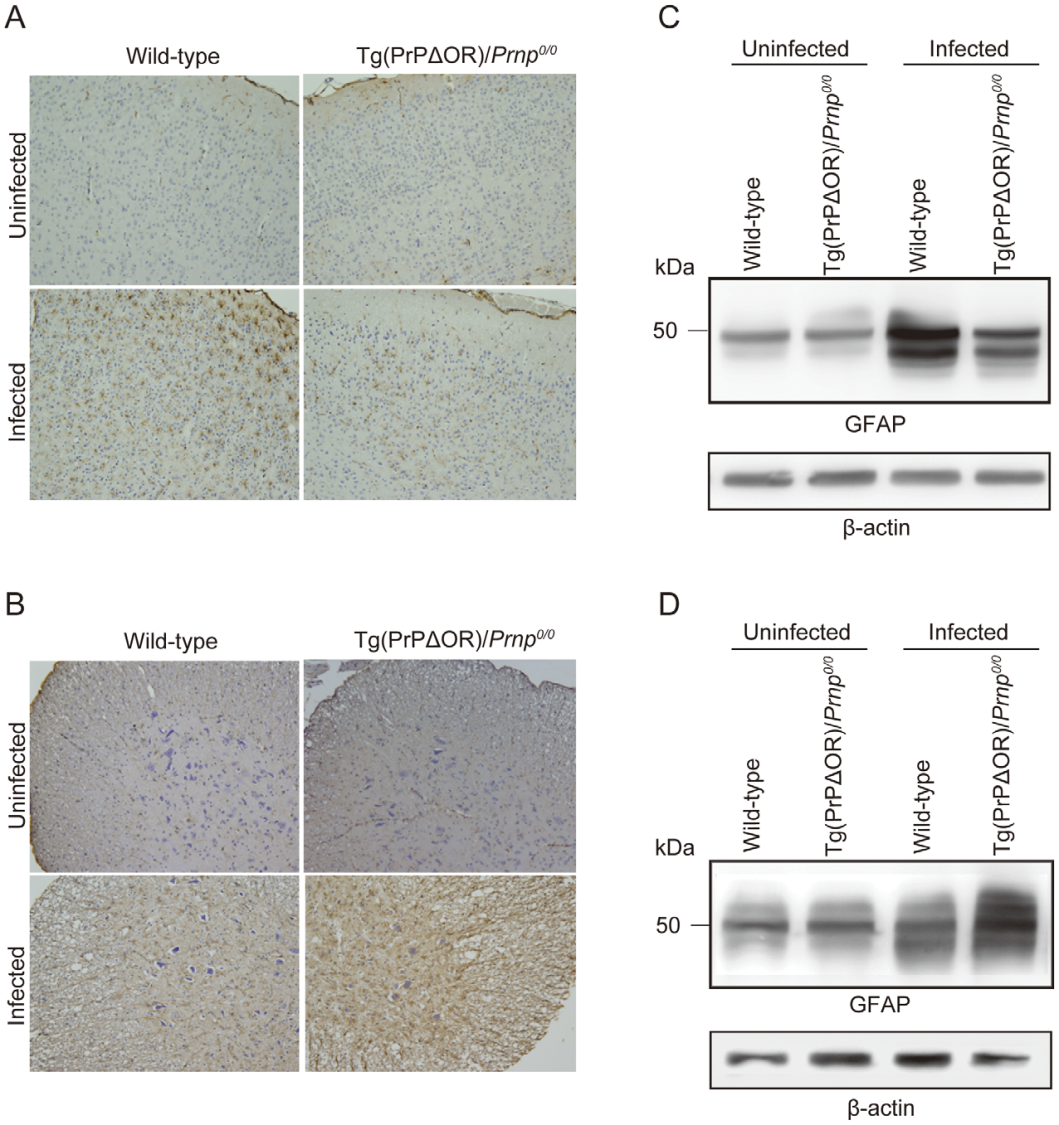
Astrogliosis in the brains and cervical cords of tg(PrPΔOR)/*Prnp^0/0^* mice infected with RML prions. The cerebral cortices (A) and cervical cords (B) of uninfected or terminally ill wild-type and tg(PrPΔOR)/*Prnp^0/0^* mice were immunohistochemically stained with anti-GFAP antibodies. The signals were milder in the brains of terminally ill tg(PrPΔOR)/*Prnp^0/0^* mice, compared to those in control wild-type mice. No decrease in the signals was observed in the cervical cords of terminally ill tg(PrPΔOR)/*Prnp^0/0^* mice. Immunoblots of the homogenates of brains (C) and spinal cords (D) of uninfected and terminally ill wild-type and tg(PrPΔOR)/*Prnp^0/0^* mice using anti-GFAP antibodies are shown. Terminally ill tg(PrPΔOR)/*Prnp^0/0^* mice expressed GFAP in their brains less than control wild-type mice. No reduction in the GFAP expression was detected in the spinal cords of terminally ill tg(PrPΔOR)/*Prnp^0/0^* mice.

We investigated brain and cervical cord sections from terminally ill tg(PrPΔOR)/*Prnp^0/0^* and wild-type mice for astrogliosis, a pathological hallmark of prion diseases, by immunohistochemical analysis using anti-GFAP antibodies. Astrogliosis was stronger in the brain and cervical cord sections from infected tg(PrPΔOR)/*Prnp^0/0^* and wild-type mice, compared to that in uninfected tg(PrPΔOR)/*Prnp^0/0^* and wild-type mice (Fig. 2, A and B). However, brain astrogliosis in infected tg(PrPΔOR)/*Prnp^0/0^* mice was slightly milder than in infected wild-type mice (Fig. 2A). In contrast, in the cervical cord sections, astrogliosis was as strong in infected tg(PrPΔOR)/*Prnp^0/0^* mice, as in infected wild-type mice (Fig. 2B). Western blotting showed consistent results. Compared to the GFAP expression in infected wild-type mice, it was mildly decreased in the brains of infected tg(PrPΔOR)/*Prnp^0/0^* mice, but not in their spinal cords (Fig. 2, C and D). We also investigated the brain sections of terminally ill tg(PrPΔOR)/*Prnp^0/0^* and wild-type mice for spongiosis. Vacuoles were similarly observed in the brains of both types of mice, scant in the cerebral cortex (Fig. S1A) but common in the hippocampus (Fig. S1B) and cerebellum (Fig. S1C). The brain sections were further immunohistochemically stained for abnormal PrP isoforms, PrP^Sc^ and PrP^Sc^ΔOR, with IBL-N anti-PrP antibodies, which were raised against the N-terminal residues 24–37, after treatment with formic acid. The immunoreactive signals were strong in the brains of both types of infected mice, compared to those in control uninfected mice, and were similarly distributed in the brains of both types of infected mice (Fig. S2).

### PK-resistant PrP, or PrP^Sc^ΔOR, in tg(PrPΔOR)/*Prnp^0/0^* Mice Infected with RML Prions

**Figure 3:**
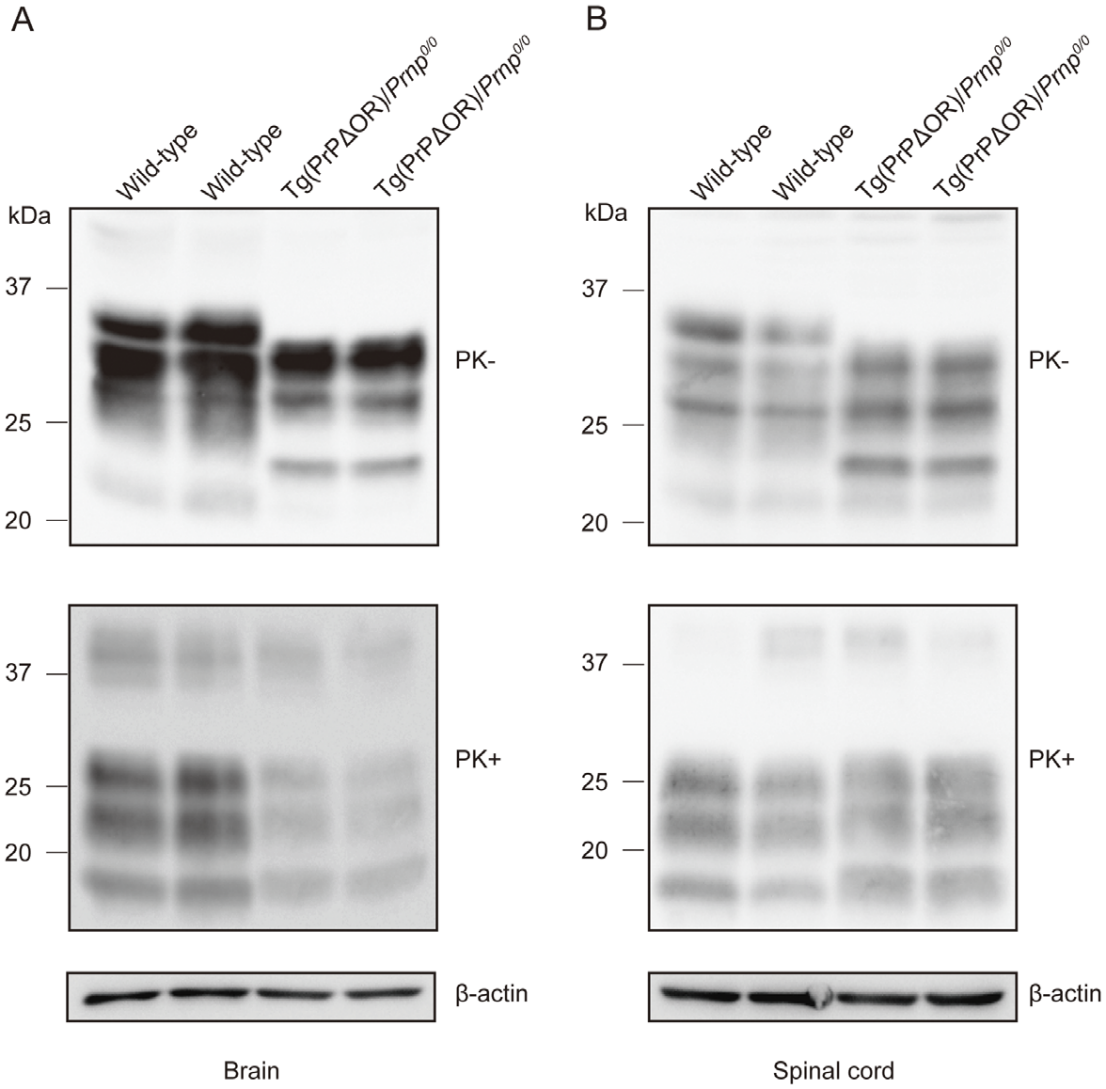
PK-resistant PrP accumulated in the brains and spinal cords of terminally ill tg(PrPΔOR)/*Prnp^0/0^* mice. Immunoblots of the two PK-treated individual brains (A) and spinal cords (B) from terminally ill wild-type and tg(PrPΔOR)/*Prnp^0/0^* mice using SAF61 anti-PrP antibodies.

We investigated the brains and spinal cords of terminally ill tg(PrPΔOR)/*Prnp^0/0^* and wild-type mice for PK-resistant isoforms, PrP^Sc^ΔOR and wild-type PrP^Sc^, respectively, using Western blotting with SAF61 antibodies. PrP^Sc^ΔOR was easily detectable in the brains and spinal cords of two individual tg(PrPΔOR)/*Prnp^0/0^* mice (Fig. 3, A and B). However, compared to wild-type PrP^Sc^ in infected wild-type mice, a reduced amount of PrP^Sc^ΔOR was detected in the brains of infected tg(PrPΔOR)/*Prnp^0/0^* mice (Fig. 3A). Western blotting of the brains showed that total PrPs were more abundant in infected wild-type mice than in infected tg(PrPΔOR)/*Prnp^0/0^* mice (Fig. 3A), despite PrPΔOR being expressed in the brains of uninfected tg(PrPΔOR)/*Prnp^0/0^* mice more than PrP^C^ in uninfected wild-type mice (Fig. 1A). This is probably due to different amounts of wild-type PrP^Sc^ and PrP^Sc^ΔOR accumulating in the brains. In the spinal cords, the amount of PrP^Sc^ΔOR in infected tg(PrPΔOR)/*Prnp^0/0^* mice was similar to that of wild-type PrP^Sc^ in infected wild-type mice (Fig. 3B).

### Prion Propagation in tg(PrPΔOR)/*Prnp^0/0^* Mice Infected with RML Prions

We also determined prion titers (LD_50_/gram of tissue) in the brains and spinal cords of terminally ill tg(PrPΔOR)/*Prnp^0/0^* and wild-type mice. To do this, we first created a standard curve between prion titers and incubation times by intracerebral inoculation of serially diluted brain homogenates of RML prion-affected mice into indicator mice. The mortalities and incubation times of the indicator mice are shown in Table 2. According to the method of Reed and Muench [14], prion titers of the homogenate were calculated as 10^8.5^ LD_50_/g. The standard curve was given by Log_10_(LD_50_/g) = 14.08−0.05X, where X is incubation time (days), 131<×<215. We thereafter intracerebrally inoculated the homogenates of 2 pooled brains and 2 pooled spinal cords from the terminally ill wild-type and tg(PrPΔOR)/*Prnp^0/0^* mice into indicator mice. The brain and spinal cord used were from the same mouse. The inoculation of wild-type brain homogenate caused the disease in indicator mice at 112±1 dpi, whereupon prion titers in the homogenate were calculated as >7.5 Log(LD_50_/g) (Table 3). However, after inoculation with tg(PrPΔOR)/*Prnp^0/0^* brain homogenate, the indicator mice succumbed to the disease with significantly longer incubation times of 150±8 dpi (Log-rank test, *p* = 0.0455), indicating that prion titers in the brains of terminally ill tg(PrPΔOR)/*Prnp^0/0^* mice were 6.7 Log(LD_50_/g) (Table 2). In contrast, in the spinal cords of infected tg(PrPΔOR)/*Prnp^0/0^* mice, prion titers were not reduced (Table 3). The spinal cord homogenates from terminally ill wild-type and tg(PrPΔOR)/*Prnp^0/0^* mice rendered the indicator mice ill at 159±8 and 142±13 dpi (Log-rank test, *p* = 0.3321), with prion titers in the homogenates being calculated as 6.3 and 7.0 Log(LD_50_/g), respectively (Table 3).

**Table 2:** Incidence rate and incubation times in wild-type ddY indicator mice inoculated with serial 10-fold dilutions of RML prions.

Dilution of inoculum (log_10_ dilution)	Incidence rate (Symptomatic mice/Total mice)	Incubation times (Mean±SD, days)
−1	6/6	131±3
−2	6/6	158±3
−3	6/6	177±6
−4	6/6	190±6
−5	6/6	215±33
−6	2/6	243, 285
−7	1/6	229
−8	0/6	
−9	0/6	
−10	0/6	

**Table 3:** Prion titers in the brains and spinal cords of terminally ill tg(PrPΔOR)/*Prnp^0/0^* and wild-type mice inoculated with RML prions.

Inoculum	Donor mouse line	Incubation times (mean ±SD, days) in indicator mice	Log-rank test *p* value	Incidence rate in indicator mice (Symptomatic/Total)	Prion titers (Log_10_/gram of tissue)
Brains (2 pooled)	Wild-type	112±1		6/6	>7.5
	Tg(PrPΔOR)/*Prnp0/0*	150±8	0.046	6/6	6.6
Spinal cords (2 pooled)	Wild-type	159±8		5/5	6.3
	Tg(PrPΔOR)/*Prnp0/0*	142±13	0.332	6/6	7.0

### The Pre-OR Region is Unusually PK-resistant in PrP^Sc^ΔOR

**Figure 4:**
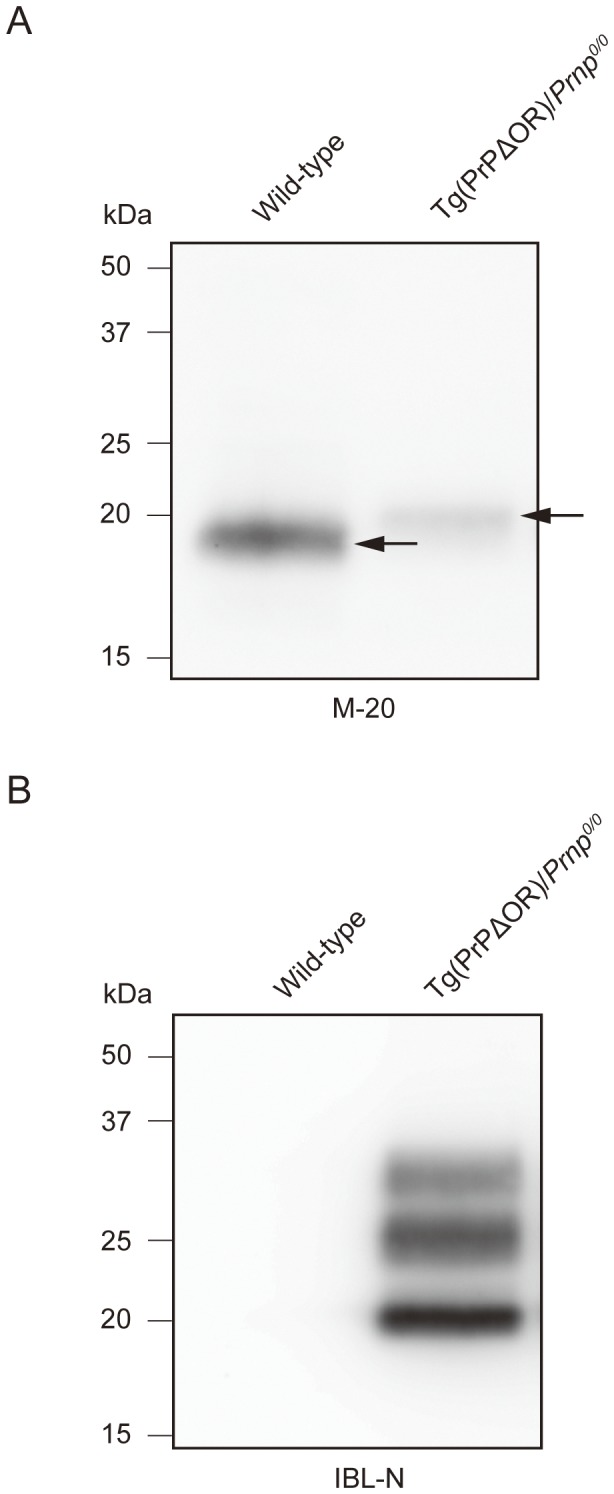
The pre-OR region of PrP^Sc^ΔOR is PK-resistant. (A) The brain homogenates of terminally ill wild-type and tg(PrPΔOR)/*Prnp^0/0^* mice were treated with PNGase F after digestion with PK, and subjected to immunoblotting with M-20 anti-PrP antibodies. The deglycosylated PK-resistant band of PrP^Sc^ΔOR was higher in molecular size than that of full-length PrP^Sc^. Arrows indicates PK-resistant deglycosylated PrPs. (B) The brain homogenates from terminally ill wild-type and tg(PrPΔOR)/*Prnp^0/0^* mice were digested with PK, and subjected to immunoblotting with N-terminus-specific IBL-N anti-PrP antibody. The IBL-N antibodies recognized the PK-resistant PrPs from PrP^Sc^ΔOR but not from full-length PrP^Sc^.

We recognized that the PK-resistant fragments of PrP^Sc^ΔOR in the brains and spinal cords appeared to migrate slightly slower than those of wild-type PrP^Sc^ on Western blotting (Fig. 3, A and B). This suggests that the PK-resistant core of PrP^Sc^ΔOR is higher in molecular weight than that of wild-type PrP^Sc^. To confirm this, we treated the PK-digested brain homogenates from terminally ill tg(PrPΔOR)/*Prnp^0/0^* and wild-type mice with PNGase F before subjecting them to Western blotting. The molecular size of the deglycosylated PK-resistant fragment of PrP^Sc^ΔOR was clearly higher than that of wild-type PrP^Sc^ (Fig. 4A). We also performed Western blotting of the brain homogenates with IBL-N antibodies raised against the N-terminal residues 24–37 of PrP. The antibody reacted with the PK-resistant fragments from PrP^Sc^ΔOR but not from wild-type PrP^Sc^ (Fig. 4B). We detected no PK-resistant fragments with molecular size >2 kDa in the brains of terminally ill wild-type mice on Western blotting using IBL-N antibodies (data not shown). Taken together, these results suggest that the entire PrP^Sc^ΔOR, including the pre-OR residues is PK-resistant, while only the C-terminal part is PK-resistant in wild-type PrP^Sc^.

### Deletion of OR Residues 51–88 Renders the Pre-OR Residues PK-resistant in Prion-infected N2a Cells

**Figure 5:**
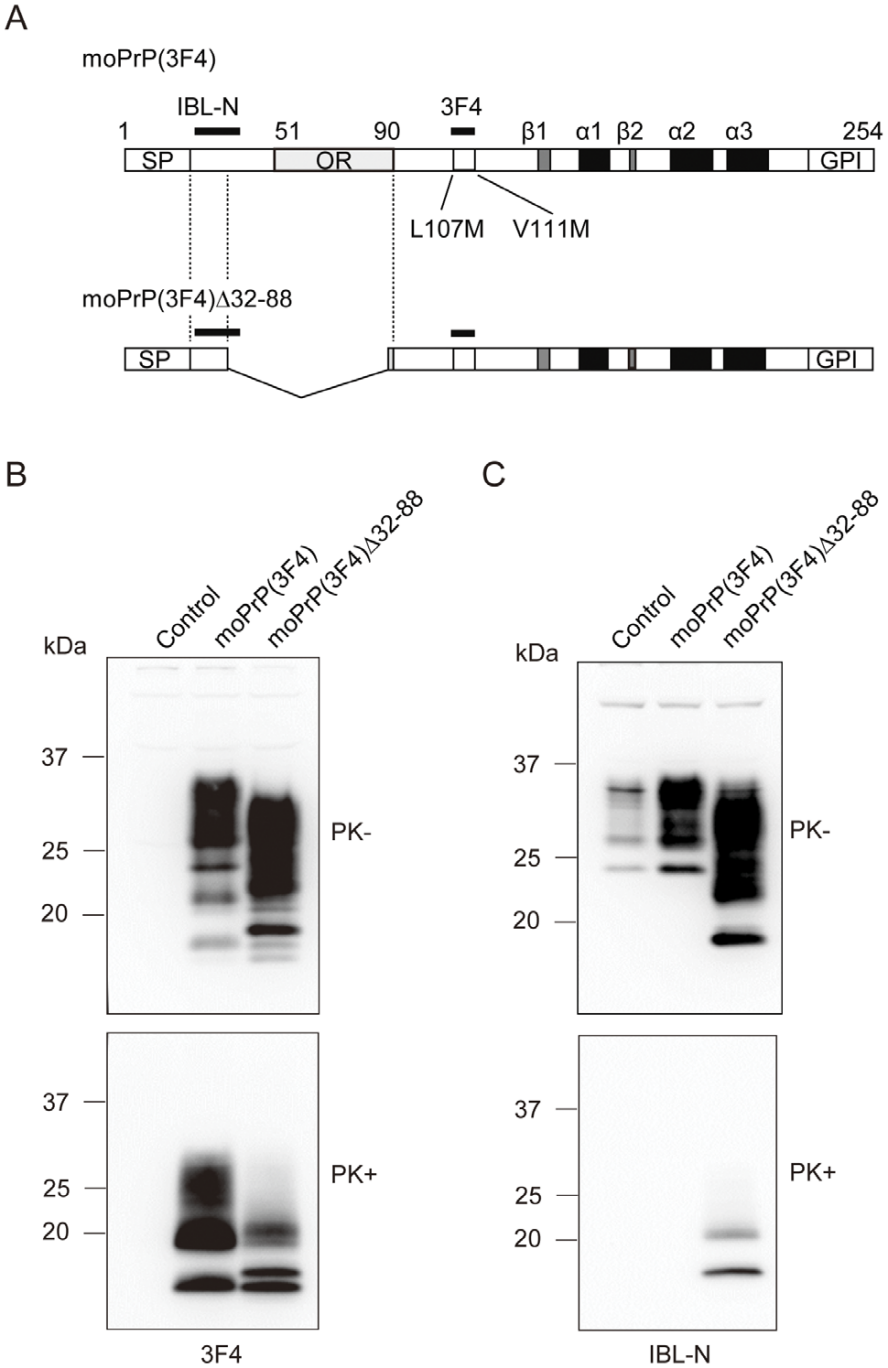
PK-resistant pre-OR residues 23–31 of PrP^Sc^Δ32–88 generated in prion-infected N2a cells. (A) Schematic diagrams of moPrP(3F4) and moPrP(3F4) Δ32–88. Arabic numbers represent the codon numbers. SP, signal peptide; OR, octapeptide repeat; GPI, GPI anchor signal; α, α-helix; β, β-strand. (B, C) Western blotting of N2aC24L1-3 cells transfected with control pcDNA3.1(+), pcDNA3.1-moPrP(3F4), and pcDNA3.1-moPrP(3F4) Δ32–88 using 3F4 (B) or IBL-N anti-PrP antibodies (C). The cell lysates were treated with PK at 5 µg/ml and then subjected to Western blotting. Both moPrP(3F4) and moPrP(3F4) Δ32–88 were converted to the PK-resistant isoforms, moPrP^Sc^(3F4) and moPrP^Sc^(3F4) Δ32–88, respectively. However, IBL-N anti-PrP antibody reacted only with the PK-resistant fragments of moPrP^Sc^(3F4) Δ32–88.

Since PrPΔ32–93 in tg(PrPΔ32–93)/*Prnp^0/0^* mice lacks the entire OR region [9], we asked whether or not the remaining pre-OR residues could become PK-resistant upon conversion. In addition, since PrPΔ23–88 in tg(PrPΔ23–88)/*Prnp^0/0^* mice has 2 residues intact in the OR region [10], we also asked whether the 2 remaining OR residues in PrPΔ23–88 could potentially block the pre-OR residues from becoming PK-resistant upon conversion. To address these questions, we constructed expression vectors encoding the 3F4-tagged mouse PrP with or without a deletion of residues 32–88, designated moPrP(3F4) and moPrP(3F4) Δ32–88, respectively (Fig. 5A), and transiently transfected them into 22L prion-infected N2a cells, designated N2aC24L1-3 cells. Using 3F4 anti-PrP antibodies, moPrP(3F4) and moPrP(3F4) Δ32–88 can be distinguished from the endogenously expressed moPrP in N2a cells. The 3F4 antibody displayed strong signals on Western blotting of the cell lysates treated with or without PK, indicating that moPrP(3F4) and moPrP(3F4) Δ32–88 were converted into PK-resistant isoforms, moPrP(3F4)^Sc^ and moPrP(3F4)^Sc^Δ32–88, respectively (Fig. 5B). Each band of non-glycosylated and mono-glycosylated moPrP(3F4)^Sc^ was single (Fig. 5B). However, they were doublet in moPrP(3F4)^Sc^Δ32–88 (Fig. 5B), suggesting that each upper band of non-glycosylated and mono-glycosylated signals could be moPrP(3F4)^Sc^Δ32–88 with the PK-resistant pre-OR region. Indeed, IBL-N antibodies reacted only with the PK-resistant fragments from moPrP(3F4)^Sc^Δ32–88, but not from moPrP(3F4)^Sc^ (Fig. 5C). The non-glycosylated band of moPrP(3F4)^Sc^Δ23–88 seemed smaller in molecular size to that of PrP^Sc^ΔOR on Western blotting (compare Fig. 4, A and B to Fig. 5, B and C). The non-glycosylated band of PrP^Sc^ΔOR was detected around 20 kDa while that of moPrP(3F4)^Sc^Δ23–88 was around 17–18 kDa (Fig. 5, B and C). This is probably because the deletion in moPrP(3F4) Δ23–88 is larger than in PrPΔOR. Taken together, these results clearly indicate that the entire pre-OR region of some moPrP(3F4)^Sc^Δ32–88 molecules are PK-resistant, and that the remaining 2 OR residues have no potential to block the pre-OR residues from becoming PK-resistant.

### Lysine Residues are Important for the Pre-OR Residues of moPrP(3F4) Δ32–88 to form a PK-resistant Structure upon Conversion in Prion-infected N2a Cells

**Figure 6:**
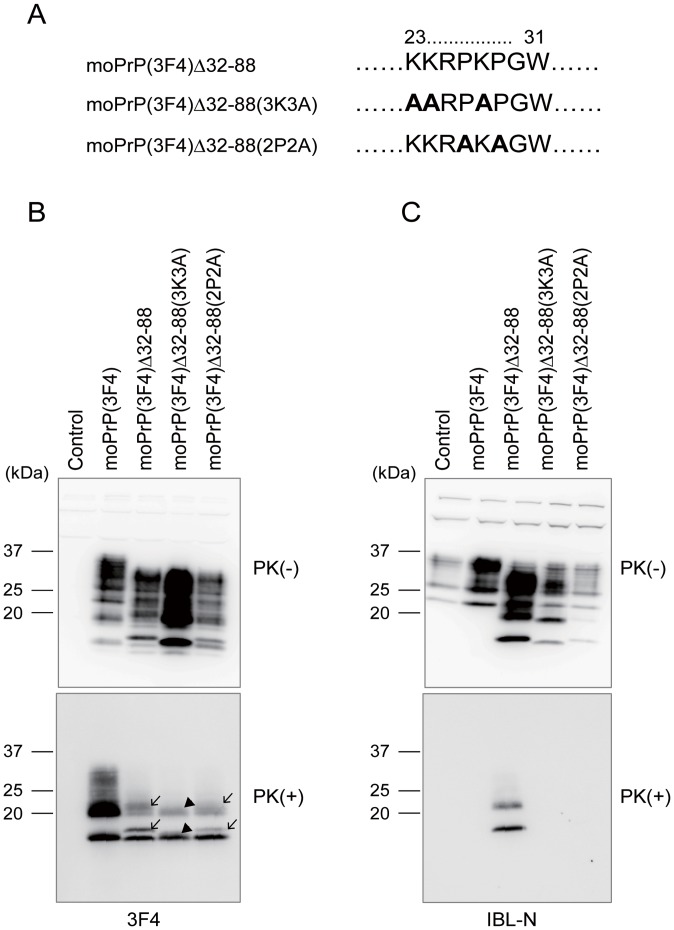
Lysine residues are important for the pre-OR residues 23–31 to form a PK-resistant structure in prion-infected N2a cells. (A) Amino acid sequences of the pre-OR residues 23–31 in moPrP(3F4) Δ32–88, moPrP(3F4) Δ32–88(3K3A) and moPrP(3F4) Δ32–88(2P2A). Bold residues indicate substituted alanine residues. (B) Western blotting of N2aC24L1-3 cells transfected with control pcDNA3.1(+) and expression vectors encoding moPrP(3F4), moPrP(3F4) Δ32–88, moPrP(3F4) Δ32–88(3K3A) and moPrP(3F4) Δ32–88(2P2A) using 3F4 anti-PrP antibodies. The cell lysates were treated with PK at 5 µg/ml. All mutant proteins were converted into PK-resistant isoforms in N2aC24L1-3 cells. The PK treatment revealed doublet non-glycosylated and mono-glycosylated bands in moPrP(3F4)^Sc^Δ32–88 (arrows), indicating that the pre-OR region of some moPrP(3F4)^Sc^Δ32–88 molecules is PK-resistant. Similar doublet bands were observed in moPrP(3F4)^Sc^Δ32–88(2P2A) (arrows). However, moPrP(3F4)^Sc^Δ32–88(3K3A) gave rise to doublet bands with the upper band migrating very closely to the lower band (arrowheads). (C) Since substitution of proline residues into alanine residues disrupted the IBL-N epitope, the PK-resistant pre-OR residues in moPrP(3F4)^Sc^Δ32–88(2P2A) failed to be visualized by IBL-N anti-PrP antibodies.

The pre-OR residues 23–31 include a very conserved positively charged region consisting of 3 lysine residues and 2 proline residues (Fig. 6A). To gain insights into the mechanism for the pre-OR residues 23–31 to be converted into a PK-resistant structure, we also constructed expression vectors encoding moPrP(3F4) Δ32–88 with a substitution of all the lysine residues or all the proline residues by alanine residues, designated moPrP(3F4) Δ32–88(3K3A) and moPrP(3F4) Δ32–88(2P2A), respectively (Fig. 6A). Western blotting with 3F4 anti-PrP antibodies revealed that both mutant proteins were converted into PK-resistant isoforms in N2aC24L1-3 cells (Fig. 6B). Non-glycosylated and mono-glycosylated bands of moPrP(3F4)^Sc^Δ32–88(2P2A) and moPrP(3F4)^Sc^Δ32–88(3K3A) were doublet (Fig. 6B). However, the upper band of the doublet was different in molecular size between moPrP(3F4)^Sc^Δ32–88(2P2A) and moPrP(3F4)^Sc^Δ32–88(3K3A). MoPrP(3F4)^Sc^Δ32–88(2P2A) gave rise to the upper band with similar molecular size to that of moPrP(3F4)^Sc^Δ32–88 (Fig. 6B). In contrast, the upper band of moPrP(3F4)^Sc^Δ32–88(3K3A) was reduced in its molecular size and migrated very closely to the lower band (Fig. 6B). The upper band of the doublet is indicative of the PK-resistant PrP molecule with the PK-resistant pre-OR residues. Thus, these results indicate that, while the pre-OR residues 23–31 are PK-resistant in moPrP(3F4)^Sc^Δ32–88(2P2A), most of them are PK-sensitive in moPrP(3F4)^Sc^Δ32–88(3K3A), suggesting that the lysine residues play an important role for the pre-OR region of moPrP(3F4) Δ32–88 to become PK-resistant in N2aC24L1-3 cells. The substitution disrupted the IBL-N epitope, resulting in loss of the immunoreativities with IBL-N antibodies (Fig. 6C). Therefore, IBL-N antibodies were not available to detect the PK-resistant pre-OR region of moPrP(3F4)^Sc^Δ32–88(3K3A) and moPrP(3F4)^Sc^Δ32–88(2P2A) (Fig. 6C).

**Figure 7:**
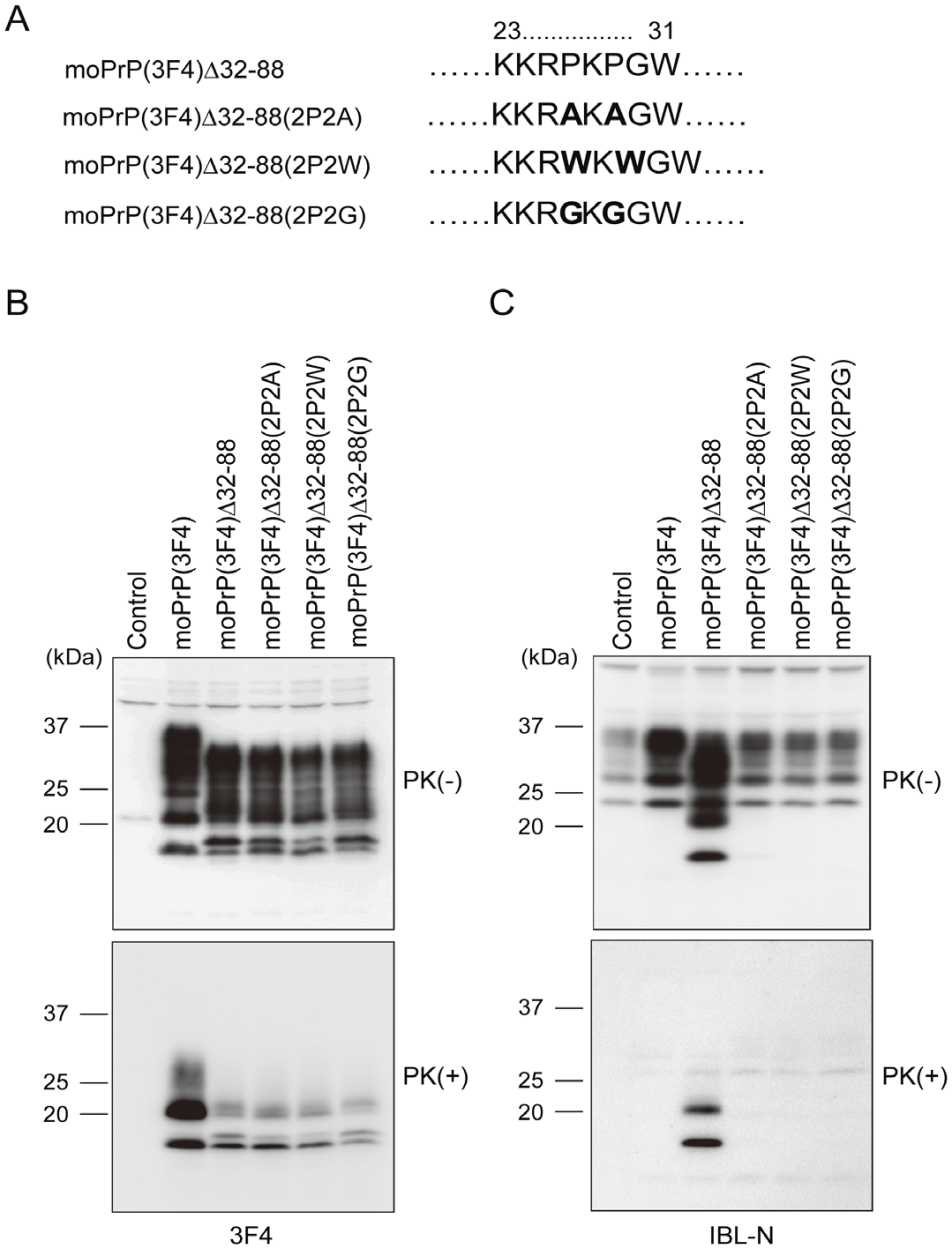
The pre-OR residues 23–31 with a substitution of the proline residues by tryptophan or glycine residues form a PK-resistant structure in prion-infected N2a cells. (A) Amino acid sequences of the pre-OR residues 23–31 in mutant proteins. Bold residues indicate substituted residues. (B) Western blotting of N2aC24L1-3 cells transfected with control pcDNA3.1(+) and expression vectors encoding each mutant protein using 3F4 anti-PrP antibodies. The cell lysates were treated with PK at 5 µg/ml. All of the mutant proteins were converted into PK-resistant isoforms in N2aC24L1-3 cells, and all of the mutant isoforms, moPrP(3F4)^Sc^Δ32–88, moPrP(3F4)^Sc^Δ32–88(2P2A), moPrP(3F4)^Sc^Δ32–88(2P2W) and moPrP(3F4)^Sc^Δ32–88(2P2G), gave rise to similar doublet non-glycosylated and mono-glycosylated bands. (C) Since substitution of proline residues into alanine, tryptophan or glycine residues disrupted the IBL-N epitope, the PK-resistant pre-OR residues in these mutant proteins failed to be visualized by IBL-N anti-PrP antibodies.

The proline residues in moPrP(3F4) Δ32–88 were further substituted for tryptophan or glycine residues in moPrP(3F4) Δ32–88(2P2W) and moPrP(3F4) Δ32–88(2P2G), respectively (Fig. 7A). Western blotting with 3F4 antibodies showed that these mutant proteins were converted into PK-resistant isoforms, moPrP(3F4)^Sc^Δ32–88(2P2W) and moPrP(3F4)^Sc^Δ32–88(2P2G), in N2aC24L1-3 cells (Fig. 7B). These PK-resistant isoforms gave rise to doublet non-glycosylated and mono-glycosylated bands that were very similar to those of moPrP(3F4)^Sc^Δ32–88 (Fig. 7B), further indicating that the proline residues are not essential for the pre-OR region to form a PK-resistant structure in N2aC24L1-3 cells. IBL-N antibodies failed to detect these mutant proteins on Western blotting since the substitutions disrupted the IBL-N epitope (Fig. 7C).

### Positively Charged Lysine Residues, Particularly those Located at Codons 24 and 27, are Important for the Pre-OR Residues 23–31 to Form a PK-resistant Structure in Prion-infected N2a Cells

**Figure 8:**
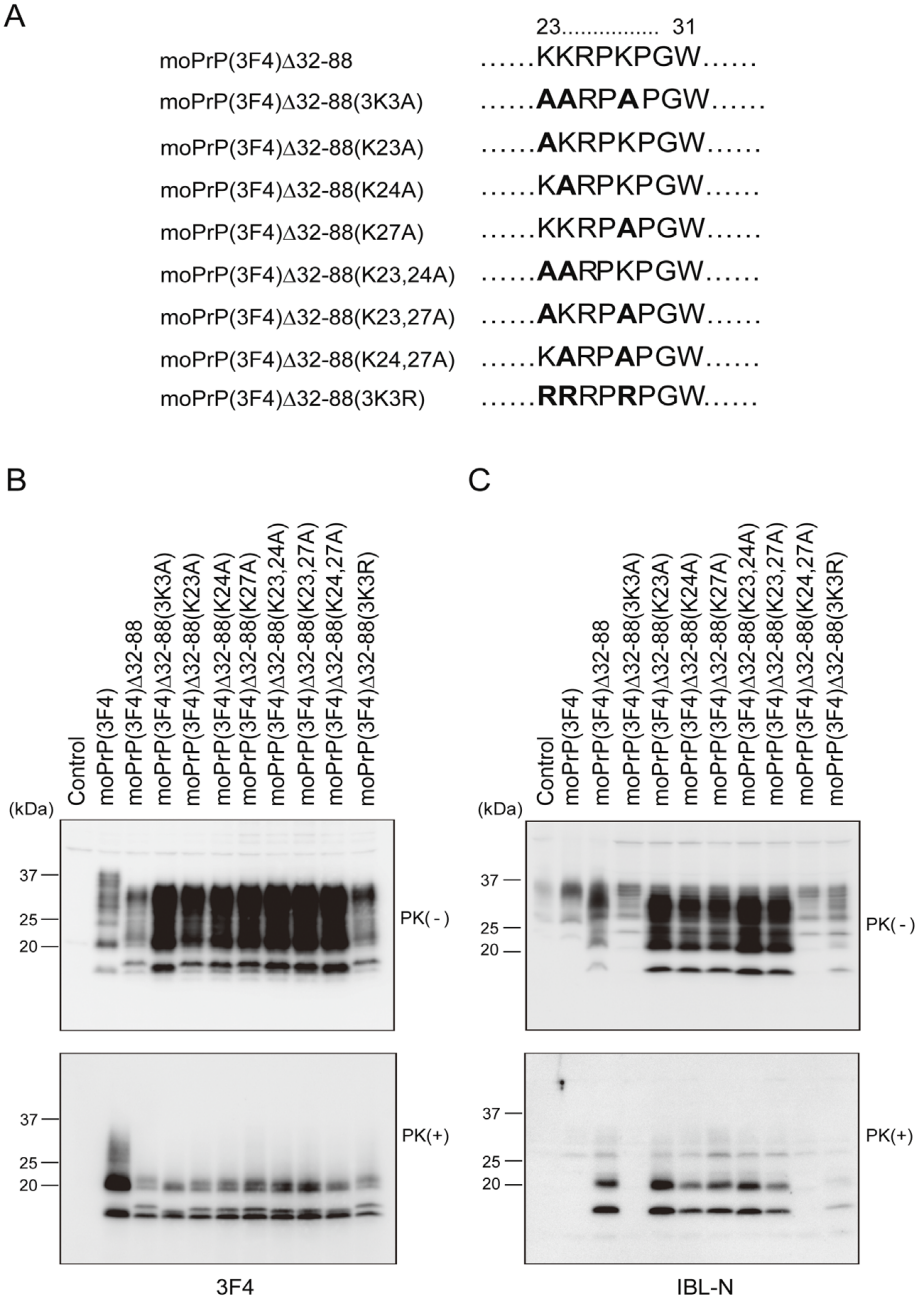
Positively charged lysine residues, particularly located at codons 24 and 27, are importnat for the pre-OR residues 23–31 to form a PK-resistant structure in prion-infected N2a cells. (A) Amino acid sequences of the pre-OR residues 23–31 in mutant proteins. Bold residues indicate substituted residues. (B) Western blotting of N2aC24L1-3 cells transfected with control pcDNA3.1(+) and expression vectors encoding each mutant protein using 3F4 anti-PrP antibodies. The cell lysates were treated with PK at 5 µg/ml. All of the mutant proteins were converted into PK-resistant isoforms in N2aC24L1-3 cells, and all of the mutant isoforms gave rise to doublet non-glycosylated and mono-glycosylated bands. The doublet bands of moPrP(3F4)^Sc^Δ32–88(K23A), moPrP(3F4)^Sc^Δ32–88(K24A) and moPrP(3F4)^Sc^Δ32–88(K27A) were similar in molecular size to those of moPrP(3F4)^Sc^Δ32–88. However, moPrP(3F4)^Sc^Δ32–88(K24,27A) gave rise to the doublet band with the upper band migrating very closely to the lower band, similarly to moPrP(3F4)^Sc^Δ32–88(3K3A). MoPrP(3F4)^Sc^Δ32–88(K23,24A) and moPrP(3F4)^Sc^Δ32–88(K23,27A) showed the upper band with an intermediate molecular size. MoPrP(3F4)^Sc^Δ32–88(3K3R) giving rise to doublet bands with similar molecular size to those of moPrP(3F4)^Sc^Δ32–88. (C) IBL-N antibodies recognized all of the PK-resistant isoforms except for moPrP(3F4)^Sc^Δ32–88(3K3A) and moPrP(3F4)^Sc^Δ32–88(K24,27A).

To gain insights into the role of the lysine residues for the pre-OR residues 23–31 to form a PK-resistant structure, one or two of the lysine residues were changed into alanine residues in moPrP(3F4) Δ32–88(K23A), moPrP(3F4) Δ32–88(K24A), moPrP(3F4) Δ32–88(K27A), moPrP(3F4) Δ32–88(K23,24A), moPrP(3F4) Δ32–88(K23,27A) and moPrP(3F4) Δ32–88(K24,27A) (Fig. 8A). Western blotting with 3F4 antibodies showed that all the mutant proteins were converted into PK-resistant isoforms in N2aC24L1-3 cells, and that all the mutant isoforms gave rise to doublet non-glycosylated and mono-glycosylated bands (Fig. 8B). MoPrP(3F4)^Sc^Δ32–88(K23A), moPrP(3F4)^Sc^Δ32–88(K24A) and moPrP(3F4)^Sc^Δ32–88(K27A) gave rise to the doublet bands with similar molecular size to those of moPrP(3F4)^Sc^Δ32–88 with the PK-resistant pre-OR residues (Fig. 8B). However, similarly to that of moPrP(3F4)^Sc^Δ32–88(3K3A) with the PK-sensitive pre-OR residues, the upper band of the doublet from moPrP(3F4)^Sc^Δ32–88(K24,27A) was reduced in its molecular size and migrated very closely to the lower band (Fig. 8B). MoPrP(3F4)^Sc^Δ32–88(K23,24A) and moPrP(3F4)^Sc^Δ32–88(K23,27A) showed the upper band with an intermediate molecular size (Fig. 8B). These results suggest that the number and position of the lysine residues might be important for the pre-OR region of moPrP(3F4) Δ32–88 to become PK-resistant in N2aC24L1-3 cells. In particular, the substitution of two lysine residues located at codons 24 and 27 affected the ability of the pre-OR region in moPrP(3F4) Δ32–88 to form a PK-resistant structure in N2aC24L1-3 cells as strongly as the substitution of all of the three lysine residues did. IBL-N antibodies recognized the upper band of the doublets from all the mutant isoforms except for moPrP(3F4)^Sc^Δ32–88(K24,27A), probably because the IBL-N epitope was disrupted in moPrP(3F4) Δ32–88(K24,27A), as in moPrP(3F4) Δ32–88(3K3A) (Fig. 8C).

We also replaced all of the lysine residues with positively charged arginine residues in moPrP(3F4) Δ32–88(3K3R) (Fig. 8A). This mutant protein was converted into moPrP(3F4)^Sc^Δ32–88(3K3R) in N2aC24L1-3 cells and the isoform gave rise to doublet non-glycosylated and mono-glycosylated bands with similar molecular size to those of moPrP(3F4)^Sc^Δ32–88 with the PK-resistant pre-OR residues (Fig. 8B). The upper band of the doublet was weakly detected by IBL-N antibodies (Fig. 8C). These results indicate that positive charges might play an important role for the pre-OR region of moPrP(3F4) Δ32–88 to become PK-resistant in N2aC24L1-3 cells.

## Discussion

Lines of evidence indicate that the N-terminal region of PrP is involved in the susceptibility of mice to prions. Tg(PrPΔ32–93)/*Prnp^0/0^* and tg(PrPΔ23–88)/*Prnp^0/0^* mice, which lack the N-terminal residues 32–93 or 23–88, respectively, developed the disease with markedly elongated incubation times after infection with RML prions [9], [10]. Moreover, *Prnp^0/0^* mice expressing PrP with further deletion in the N-terminal domain up to residue 106 from residue 32, or PrPΔ32–106, were free of the disease even after inoculation with RML prions [15]. In contrast, no extended incubation times were observed in tg(PrPΔ32–80)/*Prnp^0/0^* mice infected with RML prions [16]. PrPΔ32–93 and PrPΔ23–88 lack all or most of the OR region, respectively. However, PrPΔ32–80 still contains one intact octapeptide sequence in the OR region. This suggested that lack of the OR region from PrP could result in the decreased susceptibility to RML prions in the mice. However, in the present study, we observed no extended incubation times in tg(PrPΔOR)/*Prnp^0/0^* mice, which express PrP lacking only the OR region, after infection with RML prions. The expression level of PrPΔOR in the brain was lower than the reported level of PrPΔ32–93 or PrPΔ23–88 [9], [10]. Taken together, these results indicate that, although deletion of the OR region alone from PrP barely affects the susceptibility to RML prions, a large deletion including the OR region in the N-terminal domain could result in remarkable reduction in the susceptibility of mice to RML prions.

We observed different pathogenesis between the brains and spinal cords of terminally ill tg(PrPΔOR)/*Prnp^0/0^* mice. PrP^Sc^ΔOR and prion infectivity in the brains were lower than those in control wild-type mice. Astrogliosis in the brains was also milder than that in control wild-type mice. However, in the spinal cords, PrP^Sc^ΔOR, prion infectivity and astrogliosis were observed similarly to control wild-type mice. These results clearly indicate that, while the OR region is not essential for conversion; its deletion affects conversion taking place in the brain. Moreover, infected tg(PrPΔOR)/*Prnp^0/0^* mice developed an unusual symptom of foreleg paresis, indicating that deletion of the OR region also modifies clinical signs. These unusual phenotypes were also reported in infected tg(PrPΔ32–93)/*Prnp^0/0^* mice. This indicates that lack of the OR region from PrP induces such unusual phenotypes in mice after infection with RML prions, as observed in infected tg(PrPΔOR)/*Prnp^0/0^* and tg(PrPΔ32–93)/*Prnp^0/0^* mice. However, compared to the levels of PrP^Sc^ΔOR and prion infectivity in the brains of tg(PrPΔOR)/*Prnp^0/0^* mice, the reported levels of PrP^Sc^Δ32–93 and prion infectivity are lower in tg(PrPΔ32–93)/*Prnp^0/0^* mice [9]. Astrogliosis was easily detectable in the brains of tg(PrPΔOR)/*Prnp^0/0^* mice, but undetectable in tg(PrPΔ32–93)/*Prnp^0/0^* mice [9]. The foreleg paresis was developed at early stages in tg(PrPΔOR)/*Prnp^0/0^* mice, but only at late stages in tg(PrPΔ32–93)/*Prnp^0/0^* mice [9]. These results suggest that the effects of deletion of the OR region alone on conversion are limited, compared to those of deletion of residues 32–93 including the OR region. Therefore, the levels of PK-resistant PrP and prion infectivity were higher in the brains of tg(PrPΔOR)/*Prnp^0/0^* mice than in tg(PrPΔ32–93)/*Prnp^0/0^* mice. Consequently, astrogliosis was detectable in tg(PrPΔOR)/*Prnp^0/0^* mice but not in tg(PrPΔ32–93)/*Prnp^0/0^* mice. The onset of foreleg paresis could be associated with the amounts of PrP^Sc^ΔOR or PrP^Sc^Δ32–93 in the brain or in the spinal cord. However, the exact mechanism underlying the foreleg paresis remains unknown.

Upon the conversion of PrP^C^ into PrP^Sc^, only the 2/3 C-terminal part of PrP^C^ undergoes profound conformational changes to form the PK-resistant core of PrP^Sc^ [1]. In contrast, the N-terminal part of PrP^Sc^ remains PK-sensitive [1]. Consistent with this, we failed to detect any PK-resistant fragments with molecular size >2 kDa in the brains of terminally ill wild-type mice on Western blotting with IBL-N anti-N-terminus antibodies (data not shown). However, we found here that the entire PrP^Sc^ΔOR, including the pre-OR residues 23–50, appeared unusually PK-resistant. No data were available whether the pre-OR region of PrP^Sc^Δ32–93 were PK-resistant [9]. However, it is very likely that the region could be PK-resistant in PrP^Sc^Δ32–93 because the OR region was completely deleted in PrPΔ32–93. Indeed, we found that the entire PrPΔ32–88, including the pre-OR residues 23–31, was converted to be PK-resistant in infected N2a cells. These results indicate that the pre-OR region has a potential to undergo conformational changes to become PK-resistant upon conversion, and that the OR region usually prevents the pre-OR region from undergoing such conformational changes. We also showed that the conversion activity of the pre-OR region in PrPΔ32–88 was diminished by substitution of either all of the positively charged lysine residues or of lysine residues 24 and 27 with uncharged alanine residues, but not affected by a substitution of all the lysine residues with positively charged arginine residues. These results suggest that the positive charge at 24 and 27 residues might be important for the pre-OR region to form a PK-resistant structure when the OR region is deleted. Deletion of the authentic PK cleavage site located within the OR region might be relevant to the unusual folding of the pre-OR region. Alternatively, length of the intervening sequence between the pre-OR region and the PK-resistant C-terminal core might be a key factor to induce the unusual folding in the pre-OR region. It is also possible that, since the OR region binds to Cu^2+^ via a histidine residue [17], loss of the binding activity to Cu^2+^ might be responsible for formation of the PK-resistant pre-OR region.

The mechanism underlying the unusual phenotypes, such as brain-preferential reduction of the PrP conversion and foreleg paresis, in infected tg(PrPΔOR)/*Prnp^0/0^* and tg(PrPΔ32–93)/*Prnp^0/0^* mice remains unknown. RML prions are a strain mixture. It is thus possible that a certain specific prion strain(s) might be selected in tg(PrPΔOR)/*Prnp^0/0^* or tg(PrPΔ32–93)/*Prnp^0/0^* mice because of lack of the OR region, causing the unusual phenotypes in these mice after infection with RML prions. However, inoculation of the brain or spinal cord homogenates from terminally ill tg(PrPΔOR)/*Prnp^0/0^* mice did not induce such unusual phenotypes in wild-type mice (data not shown). Moreover, tg(PrPΔ23–88)/*Prnp^0/0^* mice were reported to show no such unusual phenotypes despite PrPΔ23–88 lacking most of the OR region [10]. These results suggest the unlikelihood of this possibility. Another possibility is that the overexpression of PrPΔOR or PrPΔ32–93 in the spinal cords might cause high accumulation of PrP^Sc^ΔOR in the spinal cords, resulting in development of the unusual phenotypes in these infected mice. However, PrP^Sc^ΔOR was accumulated in the spinal cords of infected tg(PrPΔOR)/*Prnp^0/0^* mice at a similar level to that of wild-type PrP^Sc^ in infected wild-type mice. Moreover, no foreleg paresis was reported in infected tg(PrPΔ23–88)/*Prnp^0/0^* and tg(moPrP)/*Prnp^0/0^* mice [10]. Tg(PrPΔ23–88) and tg(moPrP) mice were generated using the same cos.SHaTet expression vector system as in tg(PrPΔOR) mice [10], [12], indicating that, similarly to PrPΔOR in tg(PrPΔOR)/*Prnp^0/0^* mice, PrPΔ23–88 and moPrP^C^ are overexpressed in the brains and spinal cords of these mice. Therefore, this possibility is also unlikely. Alternatively, the unusual phenotypes in infected tg(PrPΔOR)/*Prnp^0/0^* or tg(PrPΔ32–93)/*Prnp^0/0^* mice might be due to indirect effects caused by deletion of the OR region, but not due to direct effects of deletion of the OR region. PrPΔOR and PrPΔ32–93 include the pre-OR residues 23–31 intact, whereas PrPΔ23–88 does not, suggesting that the pre-OR residues in PrP^Sc^ΔOR and PrP^Sc^Δ32–93 might be associated with the unusual phenotypes in tg(PrPΔOR)/*Prnp^0/0^* and tg(PrPΔ32–93)/*Prnp^0/0^* mice. Indeed, we showed here that the pre-OR residues of PrP^Sc^ΔOR or possibly PrP^Sc^Δ32–93 were unusually PK-resistant. The pre-OR region binds to glycosaminoglycans, the polysaccharide chains of proteoglycans, via the positively charged lysine-rich region [18], and the binding of PrP to glycosaminoglycans is important for conversion [19]. Therefore, the structurally changed pre-OR region in PrP^Sc^ΔOR and PrP^Sc^Δ32–93 might alter the binding affinity to a yet unidentified proteoglycan(s) important for conversion in the brain, resulting in disturbance of conversion in the brain. The structurally changed pre-OR region also might induce a new neurotoxic signal, causing foreleg paresis as in infected tg(PrPΔOR)/*Prnp^0/0^* or tg(PrPΔ32–93)/*Prnp^0/0^* mice. However, further studies are required to elucidate the mechanism of the unusual phenotypes in infected tg(PrPΔOR)/*Prnp^0/0^* or tg(PrPΔ32–93)/*Prnp^0/0^* mice.

There are many different groups of prion strains with strain-specific pathogenic properties [17], [20]. It is postulated that the prion strain-specific properties are enciphered in the strain-specific conformation of PrP^Sc^ [21], [22]. Since changes in the protein conformation would cause changes in PK-accessibility, PrP^Sc^s with different conformations would have different PK cleavage sites. Indeed, it was reported that two different prion strains of transmissible mink encephalopathy, HY and DY strains, produced PrP^Sc^s with different PK cleavage sites [23]. HY strain produced PrP^Sc^ with a longer PK-resistant fragment than that of PrP^Sc^ produced by DY strain [24]. We showed here that the entire PrP^Sc^ΔOR, including the pre-OR residues 23–50, appeared PK-resistant, while only the C-terminal part is PK-resistant in wild-type PrP^Sc^, suggesting that PrP^Sc^ΔOR and PrP^Sc^ might form different conformations. It might be thus interesting to characterize the biological properties of prions associated with PrP^Sc^ΔOR.

## Supporting Information

Figure S1 **Similar spongiform change in the brains of infected wild-type and tg(PrPΔOR)/** ***Prnp^0/0^*** **mice.** The brains of uninfected or terminally ill wild-type and tg(PrPΔOR)/*Prnp^0/0^* mice were subjected to HE staining. Vacuoles were scant in the cerebral cortex (A) but common in the hippocampus (B), and cerebellum (C). No specific vacuoles were observed in the brains of uninfected mice.
(TIF)

Figure S2 **Similar distribution of PrP^Sc^ and PrP^Sc^ΔOR in the brains of infected wild-type and tg(PrPΔOR)/** ***Prnp^0/0^*** **mice.** The brains of uninfected or terminally ill wild-type and tg(PrPΔOR)/*Prnp^0/0^* mice were subjected to immunohistochemistry with IBL-N anti-PrP antibodies after treatment with formic acid. The immunoreactive signals were similarly observed in the brains of both types of infected mice, but not in control uninfected mice. (A), cerebral cortex; (B), hippocampus; (C), cerebellum.
(TIF)

Table S1 Primers used in the present study.
(DOC)
