# Supplementary figures and images for: Biological and Biochemical Characterization of Mice Expressing Prion Protein Devoid of the Octapeptide Repeat Region after Infection with Prions
Source: PLoS One. 2012 Aug 21;7(8):e43540. doi: 10.1371/journal.pone.0043540 (PMC3424169; doi:10.1371/journal.pone.0043540)

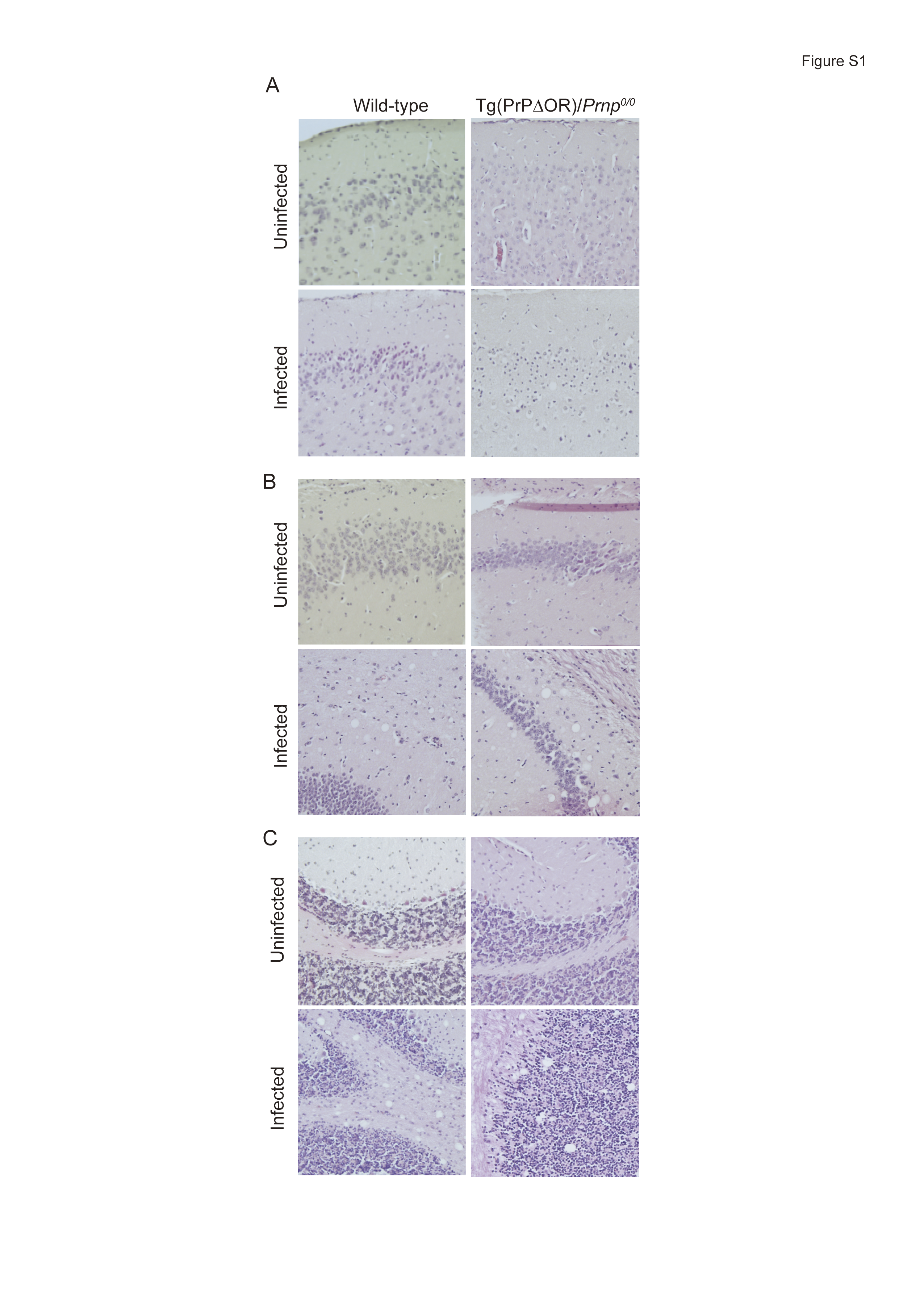

Supplement: Figure S1 — Similar spongiform change in the brains of infected wild-type and tg(PrPΔOR)/ Prnp0/0 mice. The brains of uninfected or terminally ill wild-type and tg(PrPΔOR)/Prnp0/0 mice were subjected to HE staining. Vacuoles were scant in the cerebral cortex (A) but common in the hippocampus (B), and cerebellum (C). No specific vacuoles were observed in the brains of uninfected mice. (TIF) [file pone.0043540.s001.tif]

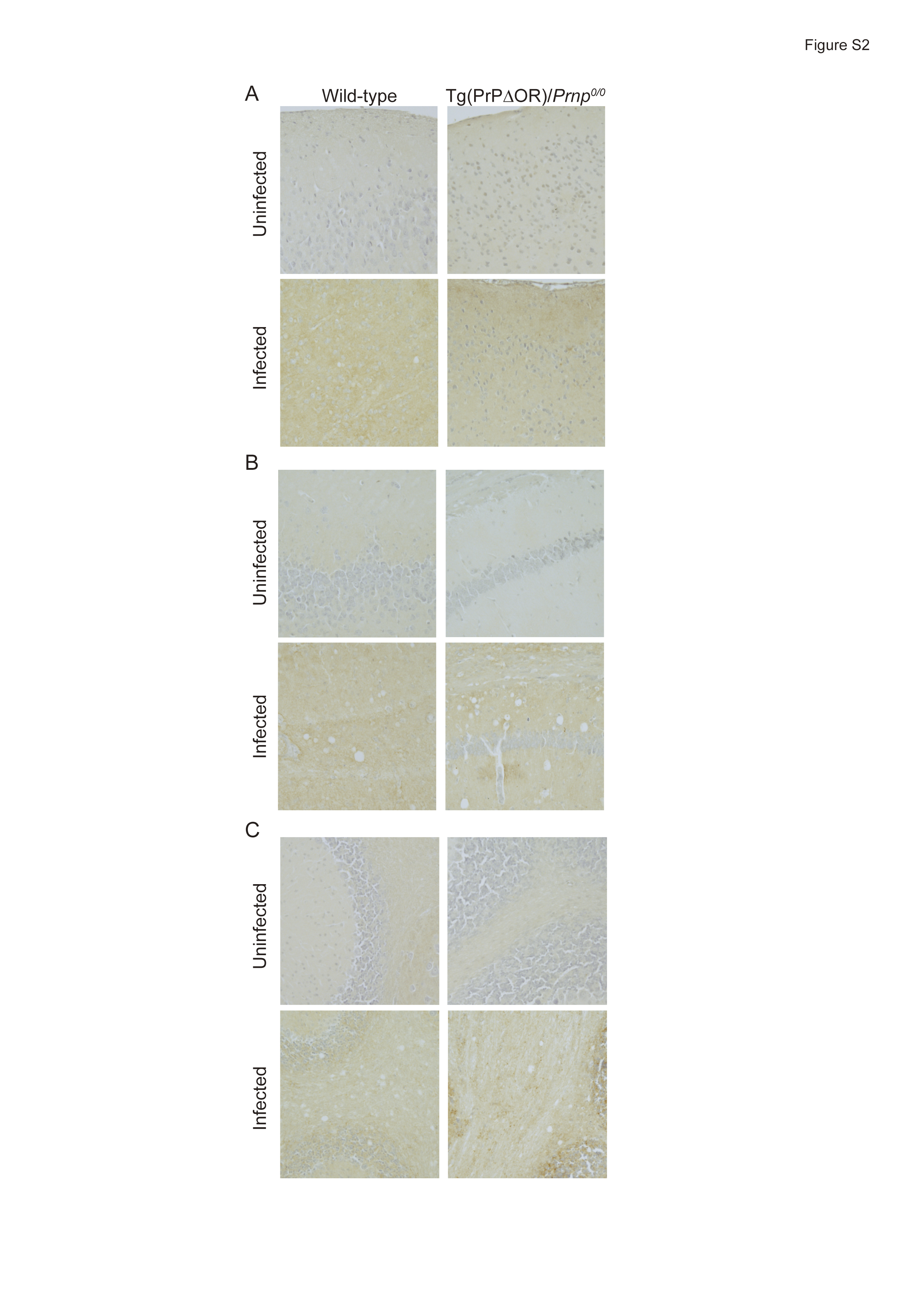

Supplement: Figure S2 — Similar distribution of PrPSc and PrPScΔOR in the brains of infected wild-type and tg(PrPΔOR)/ Prnp0/0 mice. The brains of uninfected or terminally ill wild-type and tg(PrPΔOR)/Prnp0/0 mice were subjected to immunohistochemistry with IBL-N anti-PrP antibodies after treatment with formic acid. The immunoreactive signals were similarly observed in the brains of both types of infected mice, but not in control uninfected mice. (A), cerebral cortex; (B), hippocampus; (C), cerebellum. (TIF) [file pone.0043540.s002.tif]
