# Supplementary material for: Biological and Biochemical Characterization of Mice Expressing Prion Protein Devoid of the Octapeptide Repeat Region after Infection with Prions
Source: PLoS One. 2012 Aug 21;7(8):e43540. doi: 10.1371/journal.pone.0043540 (PMC3424169; doi:10.1371/journal.pone.0043540)
Supplement: Table S1 — Primers used in the present study. (DOC) [file pone.0043540.s003.doc]

| Table S1 Primers used in the present study | | |
| --- | --- | --- |
| Primers | Sequence | Recital |
| BamHI-PrP(ATG)-S | 5’-tcggatcccgtcatc**atg**gcgaac-3’ | Underlined, *Bam*H I site; bold, start codon |
| moPrP-3F4 | 5’-ctgc**cat**gtgctt**cat**gttgg-3’ | Bold, 3F4 epitope |
| PrP(stop)-XbaI-AS | 5’-cctctagagc**tca**tcccacgatcag-3’ | Underlined, *Xba* I site; bold, stop codon |
| PrP32-88 | 5’-accccctccttggcc*ccaccctccaggctt*-3’ | Underlined, residues 89-93; italic, residues 27-31 |
| T7 | 5’-taatacgactcactataggg-3’ |  |
| BGH reverse | 5’-gctggcaactagaaggcacag-3’ |  |
| PrP(3K3A)-AS | 5’-ccaccctccaggcgctggccgcgctgcgcagaggccgac-3’ |  |
| PrP(K23A)-AS | 5’-ccaccctccaggctttggccgctttgcgcagaggccgac-3’ |  |
| PrP(K24A)-AS | 5’-ccaccctccaggctttggccgcgctttgcagaggccgac-3’ |  |
| PrP(K27A)-AS | 5’-ccaccctccaggcgctggccgctttttgcagaggccgac-3’ |  |
| PrP(K23/24A)-AS | 5’-ccaccctccaggctttggccgcgctgcgcagaggccgac-3’ |  |
| PrP(K23/27A)-AS | 5’-ccaccctccaggcgctggccgctttgcgcagaggccgac-3’ |  |
| PrP(K24/27A)-AS | 5’-ccaccctccaggcgctggccgcgctttgcagaggccgac-3’ |  |
| PrP(3K3R)-AS | 5’-ccaccctccaggccgtggccgccgccggcagaggccgac-3’ |  |
| PrP(2P2A)-AS | 5’-gttccaccctccagcctttgcccgctttttgca-3’ |  |
| PrP(2P2G)-AS | 5’-gttccaccctccgcccttgccccgctttttgca-3’ |  |
| PrP(2P2W)-AS | 5’-gttccaccctccccacttccaccgctttttgca-3’ |  |
